# Supplementary material for: Brewster quasi bound states in the continuum in all-dielectric metasurfaces from single magnetic-dipole resonance meta-atoms
Source: Sci Rep. 2019 Nov 5;9:16048. doi: 10.1038/s41598-019-52223-4 (PMC6831792; doi:10.1038/s41598-019-52223-4)
Supplement: Supplementary file 1 — Supplementary info for “Brewster quasi bound states in the continuum in all-dielectric metasurfaces from single magnetic-dipole resonance meta-atoms” [file 41598_2019_52223_MOESM1_ESM.pdf]

# Supplementary Information for

## “Brewster quasi bound states in the continuum in all-dielectric metasurfaces from single magnetic-dipole resonance meta-atoms”

Diego R. Abujetas[1], Ángela Barreda[2], Fernando Moreno[2], Juan J. Sáenz[3], Amelie Litman[3], Jean-Michel Geffrin[3*], José A. Sánchez-Gil[1*]

[1]Instituto de Estructura de la Materia (IEM-CSIC), Consejo Superior de Investigaciones Cientificas, Serrano 121, 28006 Madrid, Spain. [2]Department of Applied Physics, University of Cantabria, Santander, Cantabria 39005, Spain. [3]Aix Marseille Université, CNRS, Centrale Marseille, Institut Fresnel, Marseille, France. [4]Donostia International Physics Center DIPC, Paseo Manuel de Lardizabal 4, 20018, Donostia, San Sebastián, Spain. *Jean-Michel.Geffrin@fresnel.fr; j.sanchez@csic.es

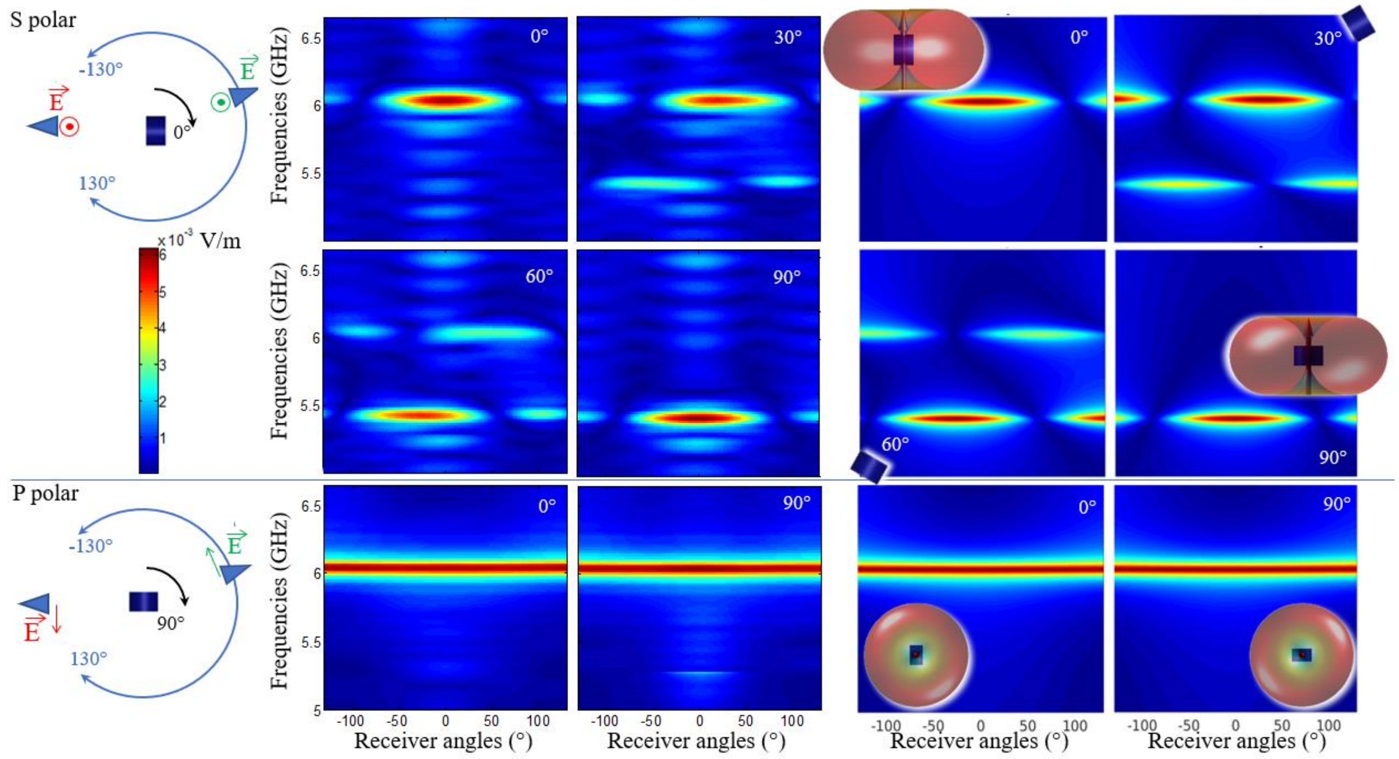


**Figure S1 | Scattering spectra of a single dielectric resonator disk** (r=3 mm and L=4 mm, ε=78+i0.05) in S (top) and P (bottom) polarizations. The scattering spectra vary in frequency and in scattering angle, the scattering plane being depicted on the left-hand-side insets. All the spectra share the same color scale, meaning that the figures can be compared in a quantitative way. The two columns on the left correspond to the experimental spectra while the two columns on the right correspond to the finite CEMD simulation. They are both plotted according to various tilts of the disk (ϕ =0°, 30°, 60°, 90°). These spectra reveal MD resonances at 5.4 GHz and 6.1 GHz, with toroidal-shaped scattering patterns as shown in the insets.

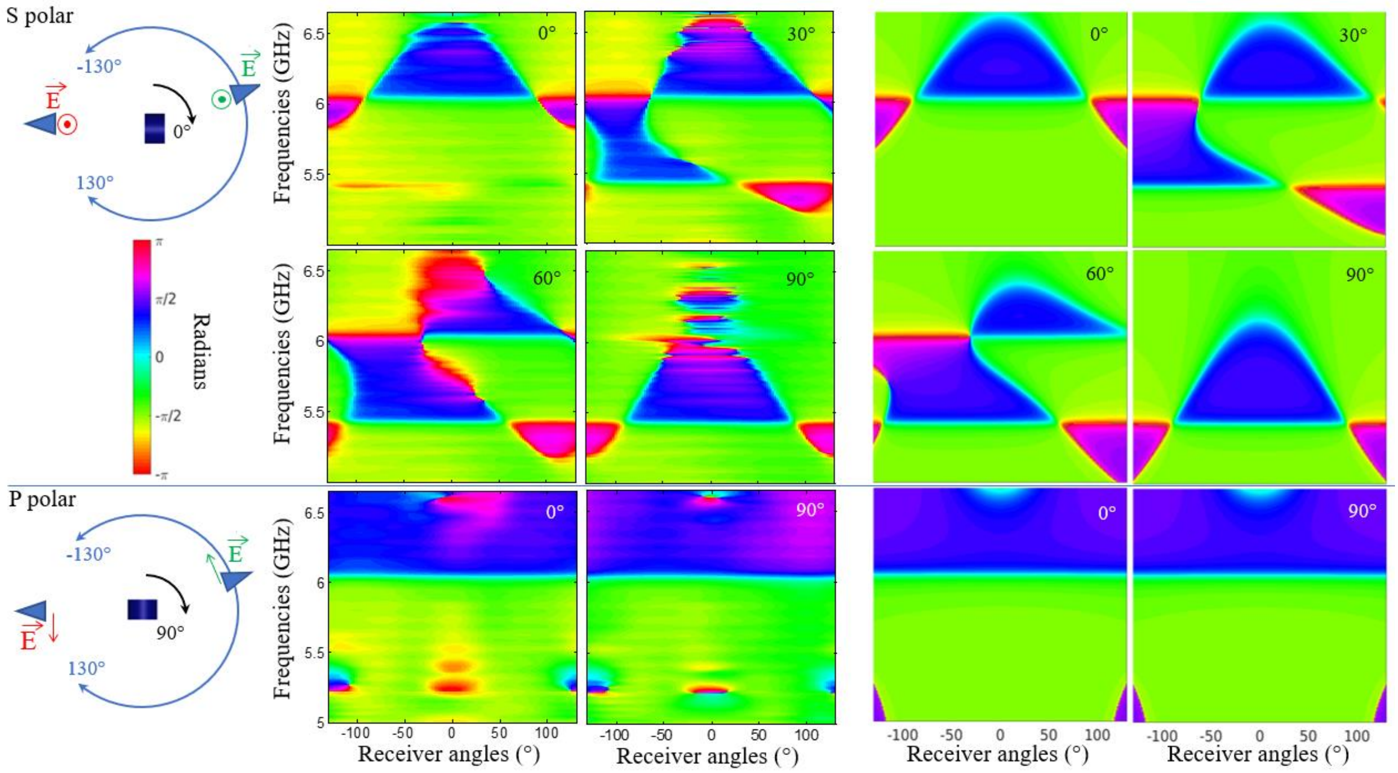


**Figure S2 | Scattering phase spectra of a single dielectric resonator disk** (r=3 mm and L=4 mm, ε=78+i0.05) in S (top) and P (bottom) polarizations. Same as in Fig. S1: the two columns on the left correspond to the experimental spectra while the two columns on the right correspond to the finite CEMD simulation.

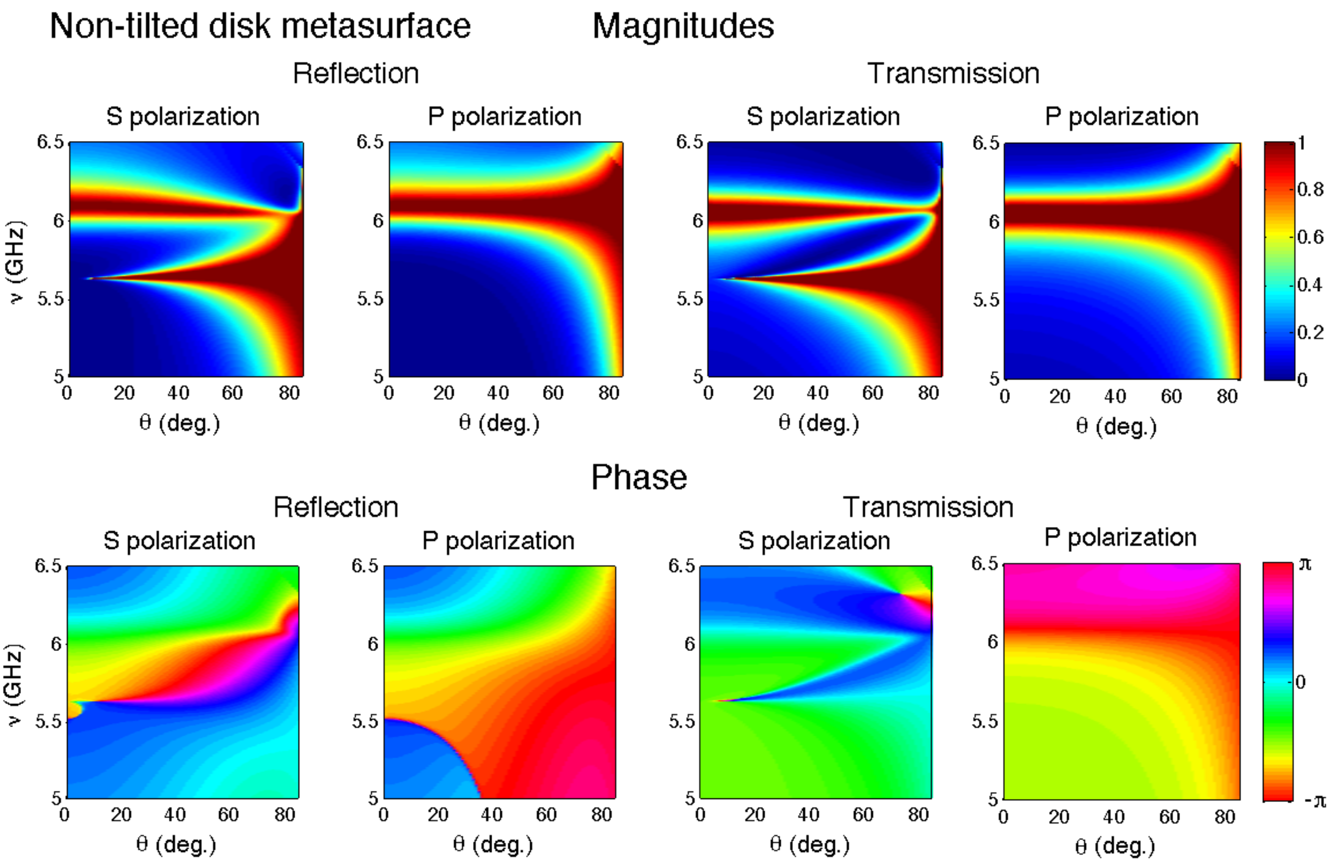


**Figure S3 | SCUFF Angularly tuned BICs in HRI disk metasurface:** Non-tilted disks, ϕ=0°. Contour maps of the SCUFF numerical calculations of the reflection and transmission intensity from an infinite square array (lattice period=12 mm) of dielectric resonator disks (r=3 mm and L=4 mm), tilted ϕ degrees with respect to the plane normal, with dielectric constant of ε=78+i0.05, as a function of angle of incidence θ (only from 0°

to 90° for the sake of symmetry) and frequency $\nu$. The reflection is calculated in the specular direction. The transmission is calculated in the forward direction and the (directly transmitted) incoming wave has been removed. (1st/2nd columns) Reflection, (3rd/4th columns) Transmission. (1st/3rd columns) S polarization, (2nd/4th columns), P polarization.

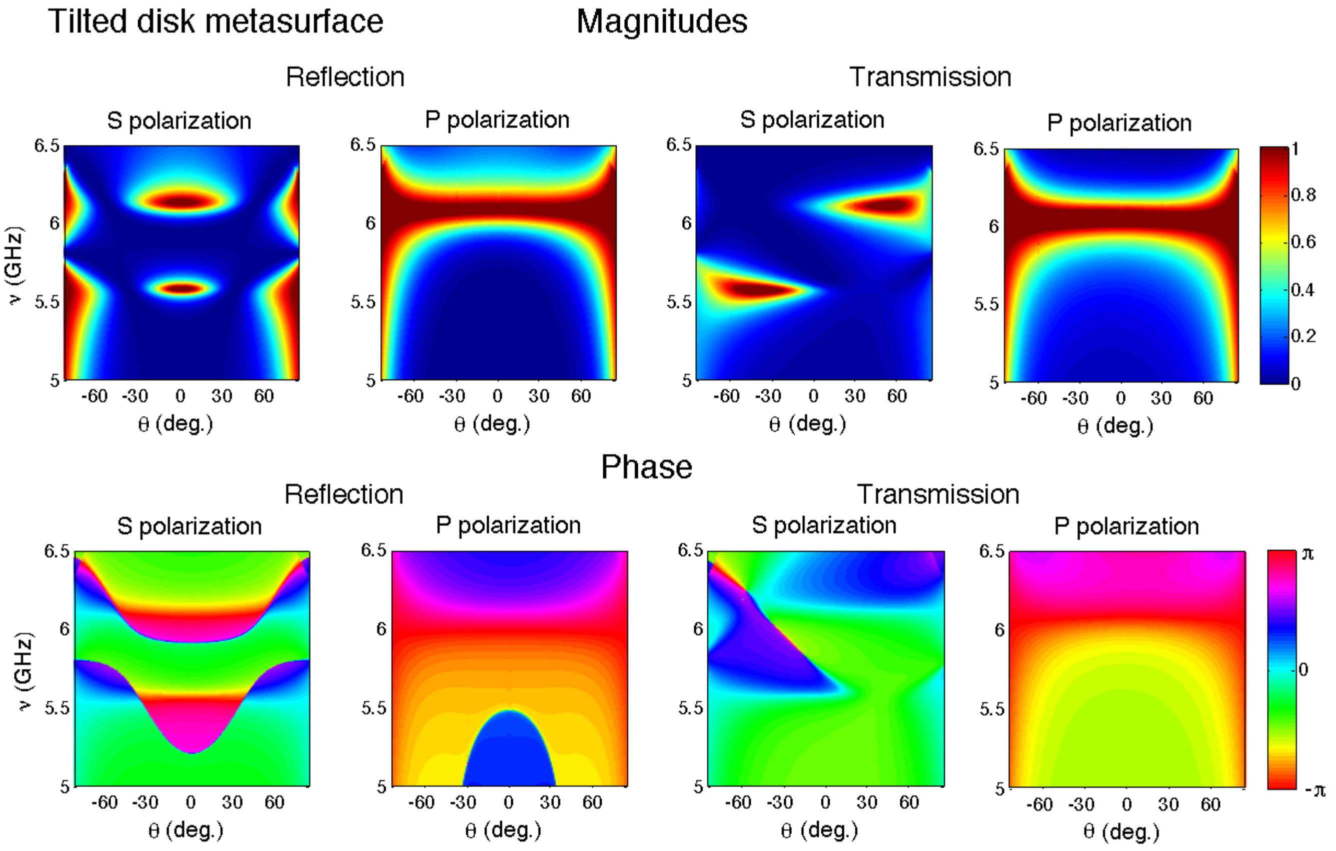


**Figure S4 | SCUFF Angularly tuned quasi BICs in HRI disk metasurface:** Tilted disks, $\phi$=-30°**.** Contour maps of the SCUFF numerical calculations of the reflection and transmission intensity from an infinite square array (lattice period=12 mm) of dielectric resonator disks (r=3 mm and L=4 mm), tilted $\phi$ degrees with respect to the plane normal, with dielectric constant of $\varepsilon$=78+i0.05, as a function of angle of incidence $\theta$ (from -90° to 90° to make asymmetry in transmission evident) and frequency $\nu$. The reflection is calculated in the specular direction. The transmission is calculated in the forward direction and the (directly transmitted) incoming wave has been removed. (1st/2nd columns) Reflection, (3rd/4th columns) Transmission. (1st/3rd columns) S polarization, (2nd/4th columns), P polarization. Note that the tilted angle is opposite to that assumed in the results shown in Fig. 5, to provide yet more evidence on the asymmetry of the disk scattering contribution to transmission.

Non-tilted disk metasurface

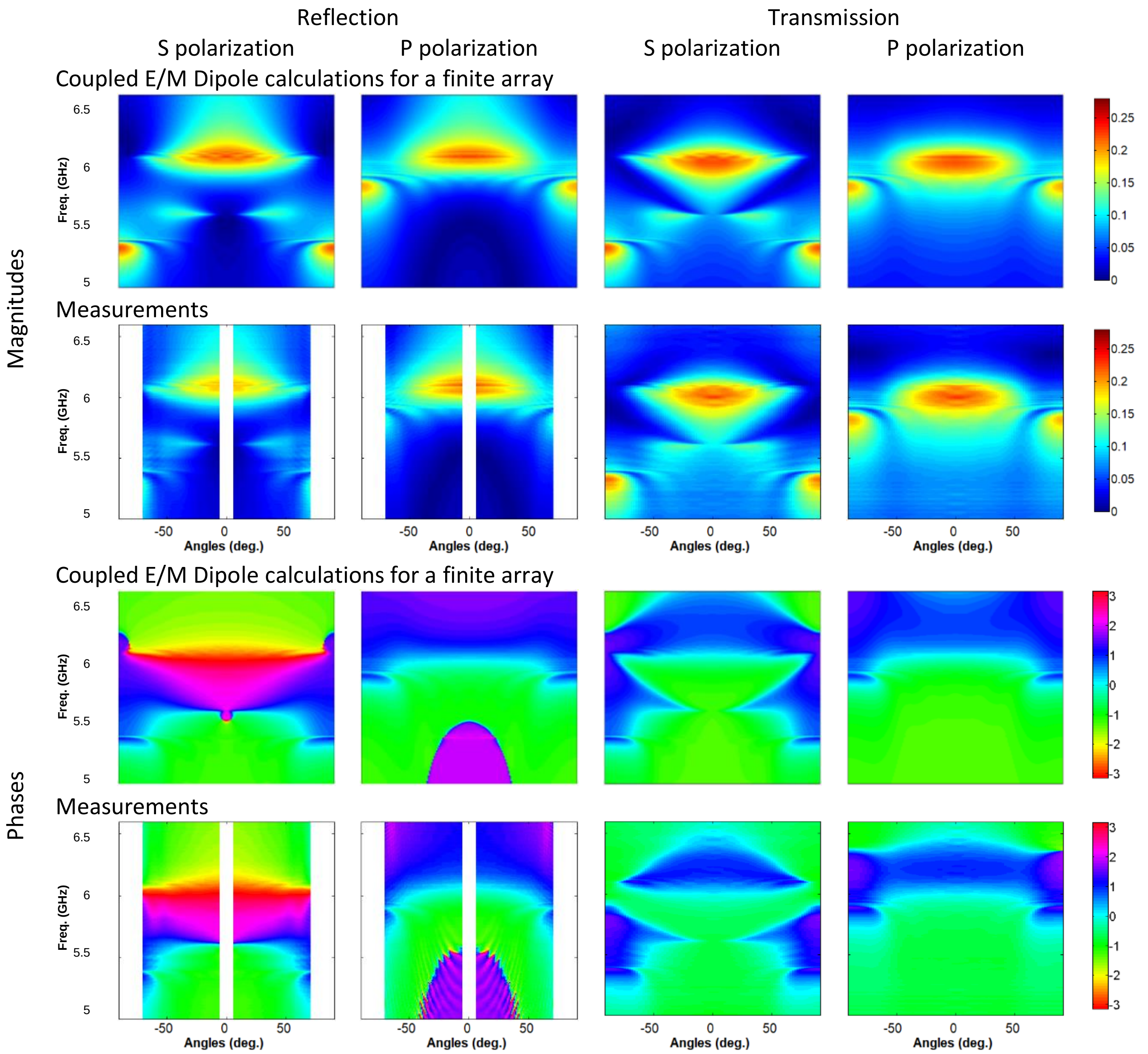


**Figure S5 | Reflection and Transmission (removing the incident wave) for a finite non-tilted (ϕ=0°) 11x11 disks metasurface** (r=3 mm and L=4 mm, ε=78+i0.05, lattice period=12 mm). The reflection and the transmission vary in frequency and in angle of incidence. The "white" areas correspond to measurement angles which are not reachable with the actual setup. The reflection is acquired in the specular direction. The transmission is acquired in the forward direction. For the reflection and the transmission, the incoming wave has been removed. All the magnitude maps share the same color scale. (1st/2nd columns) Reflection, (3rd/4th columns) Transmission. (1st/3rd columns) S polarization, (2nd/4th columns), P polarization. (1st/3rd rows) Measurements without apparent angle compensation, (2nd/4th rows) finite CEMD simulations. (1st/2nd rows) Magnitude, (3rd/4th rows) Phase.

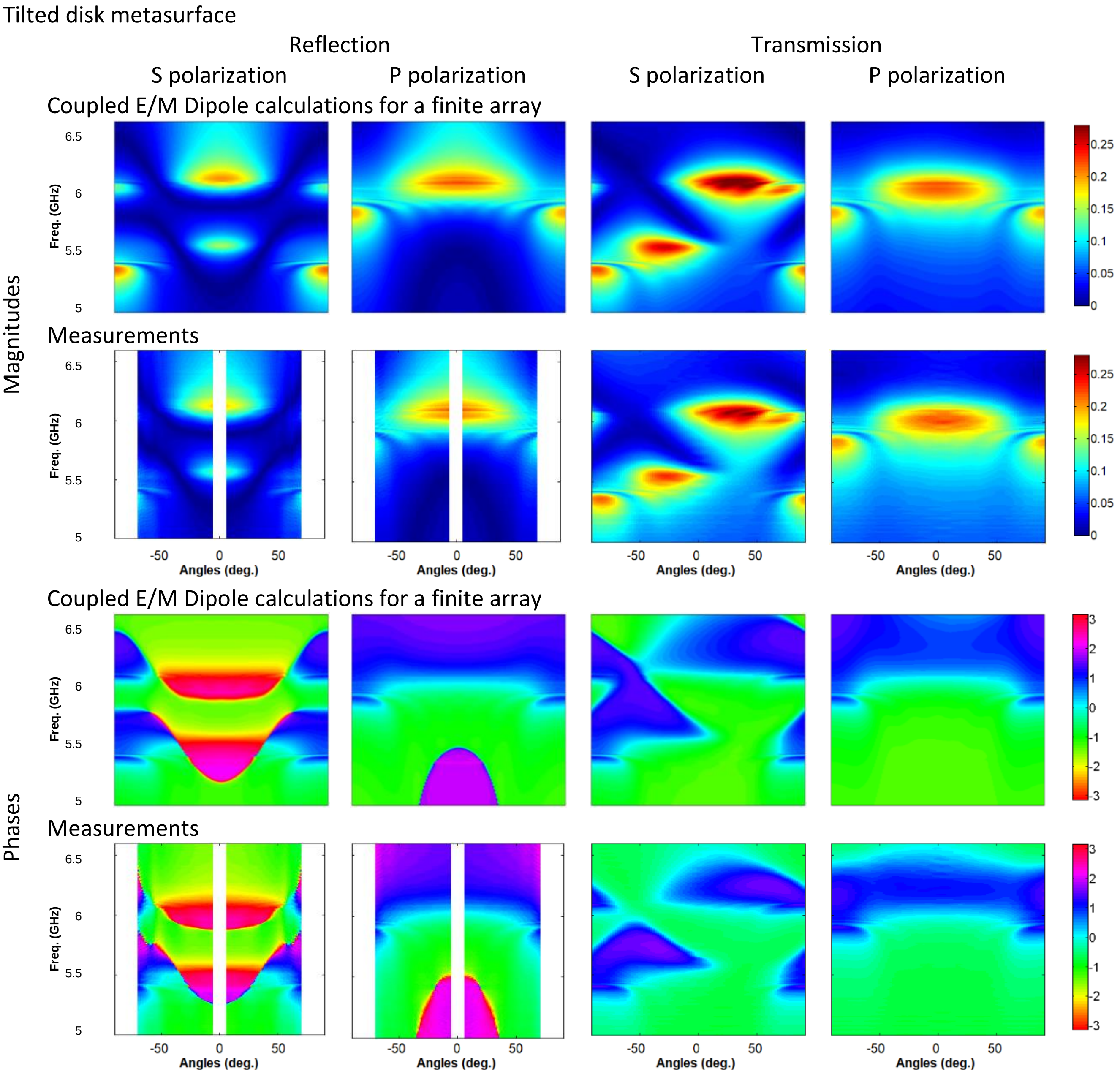


**Figure S6 | Reflection and Transmission (removing incident wave) for a finite tilted ($\phi$=-30°) 11x11 disks metasurface** (R=3 mm and L=4 mm, ε=78+i0.05, lattice period=12 mm). The reflection and the transmission vary in frequency and in angle of incidence. The "white" areas correspond to measurement angles which are not reachable with the actual setup. The reflection is acquired in the specular direction. The transmission is acquired in the forward direction. For the reflection and the transmission, the incoming wave has been removed. All the magnitude maps share the same color scale. (1st/2nd columns) Reflection, (3rd/4th columns) Transmission (1st/3rd columns) S polarization, (2nd/4th columns), P polarization. (1st/3rd rows) Measurements without apparent angle compensation, (2nd/4th rows) finite CEMD simulations. (1st/2nd rows) Magnitude, (3rd/4th rows) Phase. Note that the tilted angle is opposite to that assumed in the results shown in Fig. 5, to provide yet more evidence on the asymmetry of the disk scattering contribution to transmission.

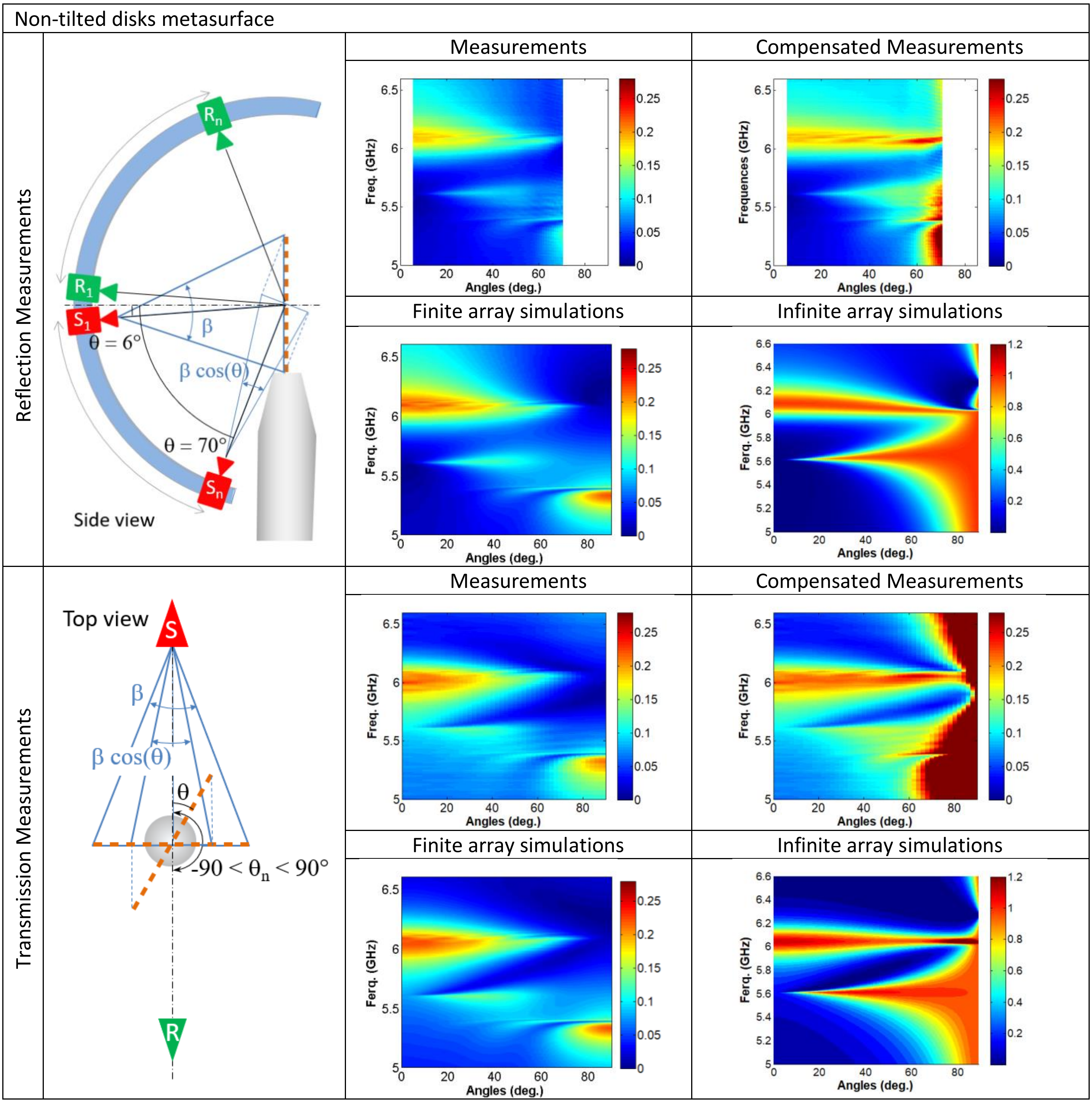


**Figure S7 | Reflection and Transmission measurement configurations description**. The left-hand-side inset describes the positioning of the emitting antenna (in red) and the receiving antenna (in green) in the reflection measurement configuration (top) and the transmission measurement configuration (bottom). In the reflection case, the emitting antenna and the receiving antenna are moving along a vertical arch in a synchronized way such that the reflection measurement is always acquired in the specular direction, while the array of disks remains fixed. In the transmission case, the emitting antenna and the receiving antenna are fixed in the azimuth plane, while the array of disks rotates on itself. In the two cases, the array of disks (in orange) is placed vertically on a polystyrene mast (transparent at microwave frequencies) located at the center of the setup. θ corresponds to the effective incidence angle, while β corresponds to the apparent angle.

For both configurations, a comparison between measurements and simulations is provided for a finite non-tilted ($\phi = 0°$) 11x11 disks metasurface (r=3 mm and L=4 mm, ε=78+i0.05, lattice period = 12mm) illuminated under S polarization. The measured magnitude maps are shown on the upper-left corner, for

various angles of incidence θ and frequencies. The associated finite CEMD simulations are shown on the lower-left corner. The measurements and the finite CEMD simulations share the same color scale. It is worth noticing the very good quantitative agreement between the two figures, both in reflection and in transmission. To compensate for the fact that in the experiments, the array is of finite dimension, a first-order angular correction factor is applied considering the apparent angle β. The corresponding Apparent Angle Compensated (AAC) measured magnitudes are shown in the upper-right corner (as "Measurements with AAC"). The associated infinite CEMD simulations are shown on the lower-right corner. The compensated measurements and the iCEMD simulations have their own color scale. Again, a very good match is visible between measured and simulated maps for the infinite case, which allow us to comfort our conclusions for finite and infinite disks arrays.

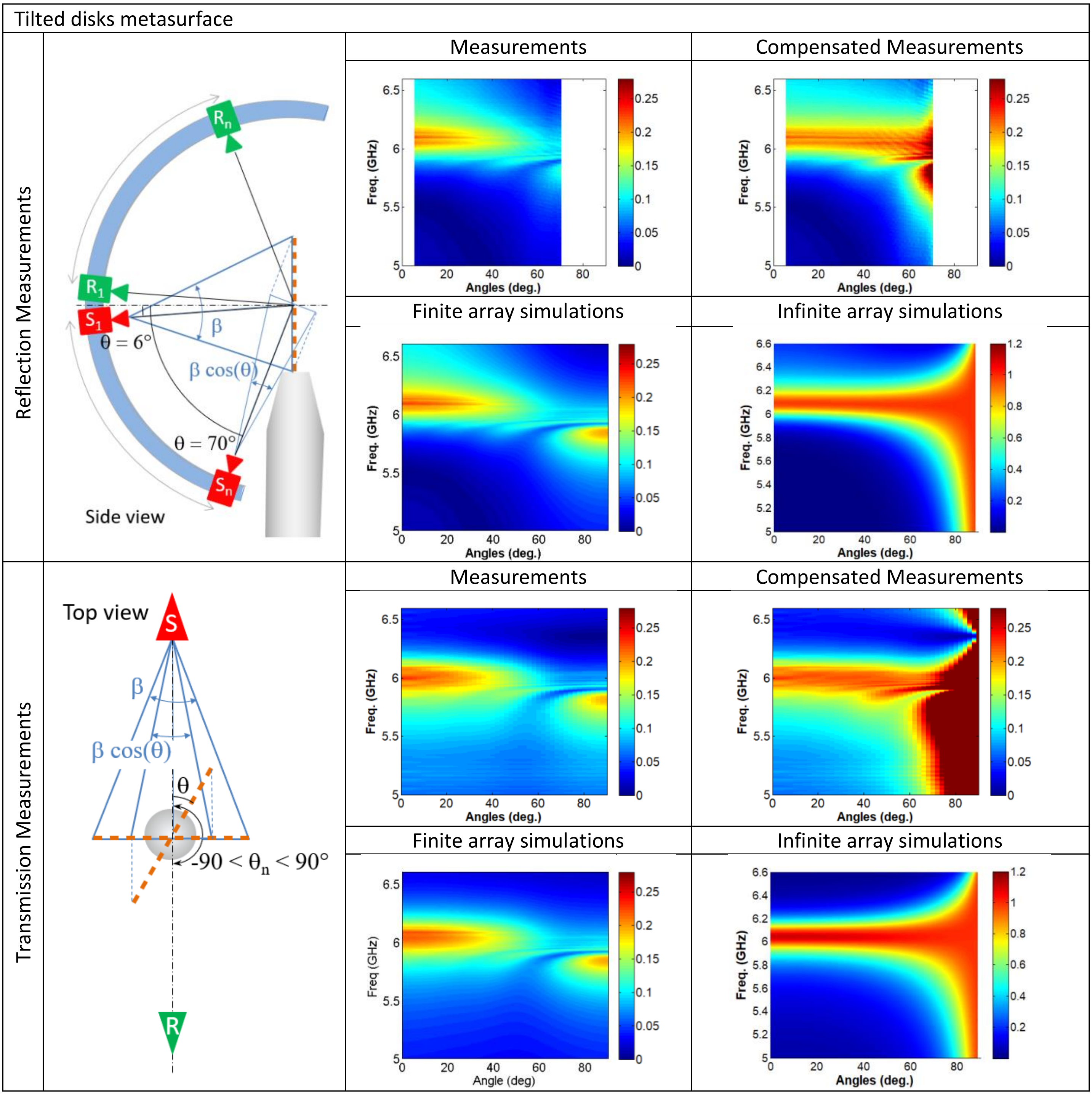


**Fig S8 | Reflection and Transmission measurement configurations description (tilted disks).** Same as in Fig. S7, but for the finite tilted (ϕ=30°) 11x11 disks metasurface (r=3 mm and L=4 mm, ε=78+i0.05, lattice period = 12mm) illuminated under S polarization.
